# “They look at us like junkies”: influences of drug use stigma on the healthcare engagement of people who inject drugs in New York City

**DOI:** 10.1186/s12954-020-00399-8

**Published:** 2020-07-31

**Authors:** Brandon Muncan, Suzan M. Walters, Jerel Ezell, Danielle C. Ompad

**Affiliations:** 1 Renaissance School of Medicine at Stony Brook University, 100 Nicolls Rd., Stony Brook, NY 11794 USA; 2 Rory Meyers College of Nursing, New York University, New York, NY USA; 3 Center for Drug Use and HIV/HCV Research, New York University School of Global Public Health, New York, NY USA; 4 Africana Studies and Research Center, Cornell University, Ithaca, NY USA; 5 Department of Epidemiology, New York University School of Global Public Health, New York, NY USA

**Keywords:** Stigma, Harm reduction, Healthcare, People who inject drugs, Syringe service programs

## Abstract

**Background:**

People who inject drugs (PWID) are a medically and socially vulnerable population with a high incidence of overdose, mental illness, and infections like HIV and hepatitis C. Existing literature describes social and economic correlations to increased health risk, including stigma. Injection drug use stigma has been identified as a major contributor to healthcare disparities for PWID. However, data on this topic, particularly in terms of the interface between enacted, anticipated, and internalized stigma, is still limited. To fill this gap, we examined perspectives from PWID whose stigmatizing experiences impacted their views of the healthcare system and syringe service programs (SSPs) and influenced their decisions regarding future medical care.

**Methods:**

Semi-structured interviews conducted with 32 self-identified PWID in New York City. Interviews were audio recorded and transcribed. Interview transcripts were coded using a grounded theory approach by three trained coders and key themes were identified as they emerged.

**Results:**

A total of 25 participants (78.1%) reported at least one instance of stigma related to healthcare system engagement. Twenty-three participants (71.9%) reported some form of enacted stigma with healthcare, 19 participants (59.4%) described anticipated stigma with healthcare, and 20 participants (62.5%) reported positive experiences at SSPs. Participants attributed healthcare stigma to their drug injection use status and overwhelmingly felt distrustful of, and frustrated with, medical providers and other healthcare staff at hospitals and local clinics. PWID did not report internalized stigma, in part due to the availability of non-stigmatizing medical care at SSPs.

**Conclusions:**

Stigmatizing experiences of PWID in formal healthcare settings contributed to negative attitudes toward seeking healthcare in the future. Many participants describe SSPs as accessible sites to receive high-quality medical care, which may curb the manifestation of internalized stigma derived from negative experiences in the broader healthcare system. Our findings align with those reported in the literature and reveal the potentially important role of SSPs. With the goal of limiting stigmatizing interactions and their consequences on PWID health, we recommend that future research include explorations of mechanisms by which PWID make decisions in stigmatizing healthcare settings, as well as improving medical care availability at SSPs.

## Background

People who inject drugs (PWID) have a higher risk of morbidity and mortality from overdose, mental illness, and transmissible infections such as HIV and hepatitis C (HCV), soft tissue infections, and endocarditis [1–5]. The higher risk among PWID compared to the general population is oftentimes attributable to poor healthcare engagement resulting from socioeconomic and structural barriers directly related to drug use or its consequences (i.e., discrimination, criminalization, unstable housing, and inability to afford care [2, 6]). From a public health standpoint, it is imperative to consider the experiences of PWID so that systemic changes can be implemented to improve the health of this medically and socially vulnerable population.

Existing literature has revealed that social stigma strongly influences PWIDs’ healthcare system engagement. The concept of stigma was formulated by Goffman in his pioneering book, *Stigma: Notes on the Management of Spoiled Identity* [7]. For Goffman, stigma was an attribute, behavior, or reputation that placed a person outside of societal norms. This alienation, which carried a negative connotation, discredited the person with the negative attribute, making society view them as less desirable. In effect, the person became marked with a “spoiled identity” [7]. Later research expanded the stigma construct beyond the individual and interpersonal levels to consider broader, macrosocial forms of stigma including structural stigma—which includes the systematic devaluation of certain identities within institutional settings such as hospitals [8, 9]. Some posited that structural stigma included institutional policies that either intentionally restricted the opportunities of, or yield unintended consequences for, certain individuals [10]; others focused on how dominant cultural norms dictated whether certain identities or statuses were socially devalued, such as the punitive responses to maternal substance use during pregnancy [11]. Of theoretical importance, Earnshaw and Chaudoir describe stigma mechanisms as related to HIV; they conclude that the interplay between enacted, anticipated, and internalized stigma influences mental and physical health outcomes [12]. Enacted stigma, as adapted from HIV to a drug use paradigm, is defined as experiences of discrimination or prejudice related to drug use felt by people who use drugs (PWUD); anticipated stigma is the expectation of future discrimination or prejudice, and internalized stigma is the acceptance of negative views and self-devaluation as a result of drug use [12]. These stigma mechanisms are of particular public health importance when experienced by PWID in healthcare settings.

Research shows that drug use stigma, and discrimination as a result of drug use stigma, adversely impacts mental and physical health [13, 14]. Compared to other forms of social stigma, stigma against drug use has been found to be greater than that against smoking, obesity, and mental illness [1, 15]. Furthermore, some drugs and drug use behaviors are more stigmatized than others, thus contributing to a hierarchy by drug type [13]. Overall, compared to non-injectors, people who inject drugs may experience higher rates of drug use and other forms of social stigma [16]. Lastly, it is important to note that several authors have independently found correlations between stigma and dangerous health behaviors, specifically demonstrating that drug use stigma is associated with increased syringe and injection equipment sharing as well as risky sexual behaviors [17–19]. These studies illustrate the importance of understanding stigma related to injection drug use, as drug use stigma impacts individuals’ behaviors, and those behaviors can increase risks for disease.

Framed within Earnshaw and Chaudoir’s three stigma mechanisms [12], we aimed to qualitatively assess PWIDs experiences with drug use stigma in healthcare and harm reduction settings.

## Methods

### Sample

We partnered with a New York City (NYC) syringe services program (SSP) that offered a comprehensive set of services including syringe exchange, safer sex supplies, pipes for smoking drugs such as crack cocaine and/or methamphetamines, HIV and HCV testing, on-site HCV treatment, opioid overdose prevention services, medical and mental healthcare counseling and referrals, case management services, various peer groups, computer and internet access, phones, and food. Self-identified PWID that used the SSP were recruited to participate in one of two studies focusing on HIV prevention. One study recruited PWID whose drugs of choice were opioids and the other recruited PWID whose drugs of choice were amphetamines. Study staff spent 1 day per week at the SSP learning about the community, building rapport with participants and staff, and conducting interviews. A total of 32 PWID, age ≥ 18 who injected drugs within the past year were identified in collaboration with the SSP staff to ensure recent injection drug use status. Informed consent was obtained from all participants. Once identified, study staff would recruit individuals for a semi-structured interview for either the opioid or amphetamine study, depending on their drug of choice. After each interview, the study staff immediately created a memo describing their observations and experiences [20]. Each participant received $40 for participating. All protocols were approved by the institutional review board at New York University.

### Interview structure

The 32 semi-structured interviews were conducted from August 2019 through February 2020. Interviews were conducted by a trained researcher and audio recorded; the interviews were conducted in a private room at the SSP by study staff (independent of SSP staff) to ensure confidentiality, and ranged in length from 30 min to 2 h. Interview questions focused on the following domains: drug/substance use history; injection history; experiences with overdose; experiences with healthcare; use of SSPs and other harm reduction services; awareness and knowledge of HCV, HIV, and pre-exposure prophylaxis (PrEP) to prevent HIV; and experiences with the criminal justice system. Demographic characteristics including age, gender, sexual orientation, education, and employment were collected at the end of each interview.

### Data analysis

Audios from the interviews were reviewed and professionally transcribed. Informed by grounded theory, constant comparison, and theoretical sampling methods [21], the data were processed and analyzed by three independent coders using Dedoose (Version 8.3.17). Relevant themes were compiled in a qualitative codebook as they became apparent in the data, and the codebook continued to change throughout the coding process as new themes emerged [22]. Codes were reviewed through dialog about the data and codebook, and a final consensus was reached among the three coders. Pseudonyms were given to participants to protect their identities when presenting quotes in this manuscript [23].

## Results

A summary of demographics is given in Table 1 and a summary of drugs injected is listed in Table 2. Briefly, of the 32 PWID interviewed, the mean age was 40.3 years (SD 8.5 years). A total of 56.3% of the participants were male, 9.4% Black, 40.6% Hispanic, 21.8% White, 18.8% multiracial, 9.4% did not specify race/ethnicity; 50% had a high school education or less, 34.3% had greater than high school education, 15.6% did not specify their educational attainment. Participants indicated injecting stimulants, opioids, or both: 34.4% injected powder cocaine, 34.4% injected crack cocaine, 34.4% injected methamphetamine, 75% injected heroin, and 18.8% injected other opioids.

**Table 1:** Demographics of PWID participants (*n* = 32)

	*n* (%)
**Age**	
18-30	6 (18.8)
31-40	9 (28.1%)
41-50	12 (37.5%)
51-60	5 (15.6%)
Mean	40.3 years; SD: 8.5 years
**Gender**	
Male	18 (56.3%)
Female	12 (37.5%)
Transwoman	2 (6.3%)
**Race/ethnicity**	
Black or African American	3 (9.4%)
Hispanic or Latinx	13 (40.6%)
White	7 (21.8%)
Multiracial	6 (18.8%)
Not specified	3 (9.4%)
**Education**	
Less than high school	6 (18.8%)
High school/GED	10 (31.3%)
Some college	9 (28.1%)
Bachelor’s degree	1 (3.1%)
Graduate school	1 (3.1%)
Not specified	5 (15.6%)

**Table 2:** Drugs injected by PWID participants in the last year*

	*n* (%)
Cocaine	11 (34.4%)
Crack	11 (34.4%)
Methamphetamine	11 (34.4%)
Heroin	24 (75.0%)
Other opioids	6 (18.8%)

*Categories are not mutually exclusive; most PWID participants injected more than one drug

Drug use stigma, in many ways, structured the healthcare experiences of PWID in this study. The majority of participants (78.1%) reported experiencing at least one form of stigma in a prior healthcare experience. The following themes were identified: (1) enacted, (2) anticipated, and (3) internalized drug use stigma. *Enacted drug use stigma* in hospitals and clinics, which contributed to the development of *anticipated drug use stigma*. Participants oftentimes explicitly attributed their stigmatizing experiences in healthcare environments to their drug use status (i.e., as opposed to their general appearance and behavior). Although we focused on all stigma mechanisms, we found only a few examples of internalized drug use stigma in our sample. A third theme emerged of participants articulating a feeling or attitude related to internalized drug use stigma—they often countered it with narratives of resistance and/or self-value. We attribute this dynamic to the positive experiences with the SSP which PWID reported. In the following sections, we illustrate each theme with quotes that highlight PWIDs’ experiences and perspectives.

### Enacted drug use stigma

Of the 32 participants, 23 (71.9%) reported some form of enacted drug use stigma including, but not limited to, discrimination (i.e., being treated negatively as a reaction to injection drug use status), and dismissive attitudes of providers at hospitals and clinics. Many participants reported direct instances in which a healthcare practitioner used language that was hurtful or had a judgmental demeanor which contributed to loss of self-worth and dignity. These participants associated poor treatment specifically to their injection drug use status. For example, when describing a consultation at a local hospital, Sophia (female, 30) explained a visit with a physician at a local outpatient clinic for a knee injury that she sustained 2 months prior. Her entire interaction with the physician changed as soon as her track marks were exposed:

Sophia: Then I waited two months and I just did a walk-in and when I met the doctor, everything was fine. As soon as I took off my coat for her to see, that was it. She went from being super nice, referring me here, to okay, maybe you should go to the emergency room…the whole entire face changed, the smile, the whole mood.

Interviewer: Because she could see your track marks is what you’re saying?

Sophia: Yeah.

Sophia attributed the recommended emergency room evaluation to the track marks (i.e., physical evidence of her injection drug use) on her body. As prior research has shown, track marks are a physical attribute that oftentimes lead to stigmatizing perspectives and behavior [24, 25]. Sophia later said that she felt insulted and devalued after the physician, with whom she had a pleasant rapport in the beginning, dismissed her, and instead recommended that she be evaluated at the emergency room.

Likewise, a number of participants described times where they felt insulted and/or disrespected by healthcare provider comments they had overheard. Carla (female, 41) recounts a time when she went into the emergency department seeking care for a persistent cough. Carla explains that she had overheard a loud discussion between providers that made her feel stigmatized and angry. She said:

I overheard them when I was in the ER, right before I was admitted last time for pneumonia. One of the doctors that was making a decision on what medication to give me said, ‘Well, I don’t think we have to worry about giving her too many benzos, look at everything she’s on.’ I’m overhearing this, and I’m like ‘Okay, you know that I can hear what you’re saying.

Continuing, Carla said that she was convinced it was her status as a drug user which contributed to the response she had received in the emergency department. Carla details a subsequent encounter at the same hospital. She had been admitted following a fall where she sustained significant leg trauma. Carla describes intense pain and difficulty moving around after having extensive reconstructive surgery. She reported that hospital staff insinuated she was injecting while in the hospital. Because of the perceived stigma experienced, Carla left that particular hospital soon after:

I mean, they accused me of using [drugs] while I was in the hospital. I was like, ‘How would I even…’ I don’t know. I didn’t even want to get into that. I transferred to another hospital right after that.

Overall, such experiences of drug use stigma from healthcare providers, often in emergency departments and the broader healthcare system, were prevalent and cause for participants to discontinue treatment or otherwise disengage with the provider.

Carla’s experience was shared among other participants who also described instances in which healthcare staff treated them differently because of their drug use. Many felt victimized, judged, and ignored in a time where they looked toward medical professionals for help. These experiences fostered apprehension about seeking future care. For example, Lucas (male, 35) explained the following occurrence during a hospitalization for opioid withdrawal, in which his complaints were dismissed as drug-seeking behavior:

You know, people tend to look at you in a certain way. Some people just stopped talking to you. Some people will just ignore you and some people will just step away from you. Well, it’s happened with the nurses. One time I was sick, and by me just being sick, they [nurses] stopped attending to me…

Maria (female, 42) framed drug use stigma as a major reason why PWID have stopped attending a local hospital-based methadone clinic. She gave her response to the enacted drug use stigma that she faced, and summarized the dehumanizing experience of being labeled as a drug user. Maria explained:

They look at us like junkies, but you know what? This junkie right here bleeds the way you bleed, have feeling the way you have feelings, love the way you love, hate the hate you hate, hold grudges the way you hold grudges. I walk the same way you walk. What’s the difference between your love and my love?…They’re [healthcare providers] so judgmental that they would literally come out and speak about you behind your back.

Below, we discuss how enacted drug use stigma leads PWID to be hesitant about seeking healthcare services (i.e., anticipated stigma).

### Anticipated drug use stigma

Of the 32 participants, 19 (59.4%) expressed some form of fear of being stigmatized or discriminated against as a result of the PWID label. In many cases, this fear led to avoidance of medical settings and providers. Francisco (male, 48) explained how previous enacted drug use stigma contributed to the anticipated stigma. He related an experience with a physician who immediately dismissed his complaints of a new-onset skin condition as drug-related, and refused to listen to the Francisco’s explanation and history. As a direct result, Francisco formed a negative perception of healthcare providers and explained that he would be reluctant to seek care in the future for fear of how he would be treated. Francisco said:

I caught scabies going to that shelter…so I used to tell the doctor. He tried to say it was the coke [cocaine]. No, the coke don’t get me like that. I’ve been doing coke for many years. Ever since I went to the shelter that’s when this rash started happening, so I’m trying to explain it to him. He’s like ‘No, it can’t be. There’s no such thing.’…But ever since then, I lost a little confidence in doctors, to be honest.

Isabella (female, 25) described feeling uncomfortable returning to her usual doctor after the physician stereotyped the appearance of a drug user and methadone maintenance therapy patient. Isabella felt stigmatized, uncomfortable, and unwilling to seek future care unless she had a true emergency because she did not want to feel devalued as a result of her injection drug use status or being in a methadone maintenance therapy program. She said:

They [physician] just start asking different questions. ‘Oh, how did that [injection drug use] come about? How did it feel like? Why? What?’ They treat you differently. ‘Well, you don’t look like the type that would usually do that.’ Well, what does that type look like? And I’m just like I’m not here for that, dude. If I don't have to even go there…I don’t.

Furthermore, while speaking about previous experiences with overdoses, Manny (male, 29) described that in the past, he had been stigmatized because of his drug use in emergency departments, and as a result, emphasized his refusal to seek medical care because of how he anticipated he would be treated by providers in the future. Manny said:

It’s different out there, man. It’s just everything’s different out there. I don’t know how to explain it. The whole aura, the way that people look at us addicts as different. You get treated bad. I’ve never been to a hospital out here for an overdose. In the hospital, I refuse to go…You definitely going to get treated differently like if you’re a drug user

Kevin (male, 43) shared the same sentiment. He had the perception that physicians have a purposefully stigmatizing attitude toward PWID. He described not seeking care because physicians will not be of help:

Interviewer: Do you think they treat people that use drugs differently?

Kevin: Yes.

Interviewer: Say more about that, what do you mean?

Kevin: Doctors look at it like for drug users, drugs are the only cure. A doctor don’t have nothing to offer an addict.

Anticipated drug use stigma was frequently attributed to previously experienced drug use stigma such as discrimination against PWID or dismissive attitudes of providers. Interestingly, the majority of participants did not report feelings of internalized drug use stigma. This is likely because of the positive experiences PWID reported at SSPs.

### Positive healthcare engagement at SSPs and resistance to internalized drug use stigma

Of the 32 participants, 20 (62.5%) reported positive (i.e., non-stigmatizing, comfortable, and accessible) experiences in terms of medical care at SSPs, particularly those services offered at the partner SSP where interviews took place. Some participants reported occasional conflict at SSPs with other clients, but in terms of accessing medical care, responses were overwhelmingly positive. PWID described the SSP as central to their daily lives—many came for the meals, used on-site technology including computers, developed social networks through group sessions, and received sterile injection equipment and medical care including HIV/HCV testing, on-site HCV treatment, and mental health counseling. The SSP oftentimes fostered self-worth, a counter-narrative to the drug use stigma experienced in hospitals and clinics. Many medical providers at SSPs had strong ties to the community and many nonmedical staff (i.e., counselors, social workers, support staff) shared lived experiences with PWID clients including previous substance use. As a result, participants described the staff, including medical and nonmedical workers, as non-judgmental, understanding, and accommodating, which made them feel more comfortable accessing and continuing care in the SSP setting. In particular, participants reported using the SSP where interviews were taking place for HCV and HIV testing and treatment.

Carla, who reported several previous instances of drug use stigma at a local hospital, spoke about how both she and her husband were able to get tested for HCV at the SSP. Although it was found upon re-testing that Carla had cleared the HCV, her husband was diagnosed with HCV and was offered treatment at the SSP. She emphasized the SSP environment had been accessible and non-stigmatizing, and that she would like to get involved in other services offered such as peer groups at the SSP because of the positive atmosphere. Carla said:

Carla: They’ve helped tremendously, especially with having clean needles. I did have hepatitis C. I was going to get treatment and they said ‘you no longer have it,’ so it was awesome.

Interviewer: You did the test right here?

Carla: Yeah. My husband still has it. He’s getting treatment.

Interviewer: Does he come here too?

Carla. Yeah…I’m grateful that there’s a place to come and get clean needles, things like that.

Kira (female, 41) contrasted the non-stigmatizing attitudes of providers in this environment with those of providers in the larger hospital/clinic systems. She says that the SSP environment makes PWID feel safe, and that it would be an accessible and effective place to access medical care. Kira said:

I feel like I’m not being judged here and things like that. A lot of times people avoid hospital settings for whatever reason, whether they’re scared of doctors or they just don’t want to know…they just shut down. I think in a place like this [SSP], they leave themselves open.

Francisco summarized many PWID’s opinion about using SSPs instead of other healthcare settings when he said:

They know where we’re coming from. They should build more places like this.

Francisco recognized that the accepting environment of his local SSP and the absence of enacted drug use stigma lessened PWIDs’ anticipated sigma and therefore minimized their fear and reluctance to seek care at these facilities.

## Discussion

PWID in our study highlighted a variety of pronounced and influential experiences with drug use stigma in the broader healthcare environment. Whether a result of enacted stigma (including discriminatory or injurious actions of healthcare providers, and/or dismissal of concerns secondary to injection status) or anticipated stigma, PWID reported that stigmatizing experiences influenced their health and healthcare seeking in some form. These findings echo PWID perspectives in qualitative studies conducted in other locations, including Boston (MA), Providence (RI), and Fresno (CA), where research has identified healthcare stigma among PWID and noted the importance of harm reduction settings in the day-to-day experiences of PWID [2, 26]. We speculate that the SSP environment may improve PWIDs self-worth and sense of belonging, providing them with the resources they need to express their circumstances and identity. Further research is needed to understand PWID perspectives on how they balance the risk of experiencing drug use stigma with honest health-related dialog in healthcare settings.

Study participants found healthcare more accessible and less stigmatizing at local SSPs that offered medical services; participants said they felt more comfortable because staff were aware and respectful of the struggles of PWID, likely because SSP staff shared many lived experiences with their clients. The unique relationships between SSP staff and PWID are likely key to SSP success in providing non-stigmatizing medical care. In many cases, participants were able to access HIV and HCV testing and treatment as well as mental health counseling in a comfortable and accepting environment, which was reported to be conducive to care continuity. In large urban areas such as NYC, PWID may elect to find medical advice, consultation, and treatment at SSPs; however, SSP services may not be readily available in smaller communities, or even in other metropolitan environments. Furthermore, other SSPs may not be as robust as the one assessed in this study. PWID living in rural areas may be particularly vulnerable given the general lack of healthcare and social services in these communities and other endemic issues such as limited transportation and social norms against drug use and other perceived “deviant” behaviors. In addition, SSPs in rural areas may operate on tighter financial margins and thus may not have the capacity to provide robust medical services, creating a burden for PWID who do not feel comfortable going to hospitals for care. Previous studies have shown public resistance to SSP implementation, which could limit availability of services as well [27]. In these cases, PWID may not be able to access medical care in comfortable, non-stigmatizing environments, which may contribute to internalized drug use stigma and poorer health outcomes [24, 28].

Internalized drug use stigma has been associated with poor health outcomes and healthcare system engagement [28]. The lack of reported internalized drug use stigma in our study may be related to PWIDs’ counterbalancing positive SSP experiences. Positive healthcare engagement in SSP settings established feelings of value and self-worth for PWID, which could have played a role in mitigating internalized drug use stigma following negative experiences at hospitals and local clinics [27]. Dignity may play a role in explaining this phenomenon as previous work has found that denying the dignity of vulnerable populations (i.e., PWID) may predispose them to increased stress and risky behaviors, which may in turn lead to poor health outcomes [29]. Our finding of the critical importance of SSPs in the delivery of effective, non-stigmatizing medical care has important public health policy implications and should be investigated further as a mode of healthcare delivery for vulnerable populations like PWID.

Previous research has given validity to PWID perceptions of being stigmatized in the healthcare system: many authors have concluded that healthcare professionals oftentimes hold negative perceptions or implicit biases against persons with substance use disorders, and these views may contribute to poorer patient-provider interactions [30–33]. More specifically, once a negative perception on the part of providers is formed, those patients with substance use disorders may become stigmatized and alienated in the healthcare setting. This enacted drug use stigma produces a reactive avoidance of seeking care (through anticipated stigma) and can contribute to rising morbidity and mortality from associated disease [34]. More research is needed, therefore, to understand how prevalent drug use stigma against PWID is, among healthcare providers, if bias varies by sub-populations of PWID, and how these perspectives impact both acute and longitudinal care for PWID.

Overall, our data provide insights into enacted and anticipated drug use stigma against PWID in New York City. The majority of participants opined that injection status was an explanation for being stigmatized while accessing care. PWID experiences in the healthcare system may have fostered anger, frustration, and distrust toward healthcare providers, and contributed to a reluctance to engage in medical care until a health problem had effectively peaked and became emergent. Consequent prolongation of time-to-diagnosis and time-to-treatment of common conditions seen among PWID (infection, overdose, mental illness) present a key public health dilemma that should be addressed on a policy level. One policy that could benefit PWID is increasing the availability of SSP services where feasible. Although there are political and economic constraints that may limit expansion of SSP services, research has shown that PWID trust community-based organizations more than traditional healthcare settings [2] and that peer groups improve narratives of internalized drug use stigma [25]. SSPs, as community-based, PWID-oriented venues may therefore be beneficial both in improving access to medical care for PWID and in mitigating drug use stigma. Furthermore, we recommend strategies and interventions to minimize drug use stigma in traditional healthcare settings, such as implementation of programs to improve awareness of, and educate about, substance use disorders and harm reduction among healthcare providers, community members, and policymakers [1, 31]. In particular, programs aiming to teach medical students, physicians, law enforcement officers, and counselors have shown to be effective in reducing drug use stigma and are suggested as structural interventions, particularly if PWID can be active members in the process by voicing their perspectives and discussing their concerns as part of the teaching initiative [25]. Lastly, the impact of dignity should also be considered in the attempt to lessen drug use stigma and improve care for PWID. As adapted to substance use among PWID from an HIV paradigm, many stigmatized individuals’ dignity is attacked as a result of the drug use label [29]. As such, political campaigns and social movements aiming to disband attacks on PWIDs’ dignity, and to recognize and understand substance use disorders as medical entities may improve narratives of internalized drug use stigma among PWID, improve access to non-stigmatizing care, and ultimately may lead to improvements in health for PWID [1, 29, 31].

### Limitations

Recruitment of participants and data collection was carried out at an established SSP in NYC, which offered a comprehensive array of services. NYC has many accessible harm reduction facilities, unlike most cities in the USA [35, 36]. Therefore, our findings may not be generalizable to other PWID populations and treatment environments, particularly those outside of NYC and other areas that are not well-resourced. Moreover, PWID in NYC that do not use SSPs may have different experiences than the ones we report here. Lastly, we did not collect data on smoking and/or tobacco use, which is also a stigmatized behavior in healthcare settings [15], and may have provided another mechanism for stigma among our sample.

## Conclusion

Given that PWID represents a group with increased morbidity and mortality from many causes [2–5], it is imperative to understand PWIDs’ attitudes and histories with respect to medical care provision and harm reduction. This study aimed to add to the body of qualitative literature on drug use stigma experienced by PWID in relation to the US healthcare system. Our data highlight formative experiences among PWID in NYC’s healthcare landscape; namely, enacted and anticipated stigma in local hospitals and clinics because of drug injection status, as well as positive healthcare engagement experiences at a local SSP. Our findings coincide with those of other authors and point to notable drug use stigma against PWID in NYC healthcare settings. We contribute to the literature by adding that SSPs were often regarded as safe and accepting sites for medical care provision and that these positive experiences at SSPs may mitigate internalized drug use stigma. These findings should be considered when developing future policies regarding healthcare services available to PWID; specifically, we should consider how to embed healthcare and other treatment services within established harm reduction organizations that provide non-stigmatizing safe spaces for PWID (e.g., as in the case of a “medical home”). Moreover, there is also a clear and immediate need to implement structural changes to limit stigmatizing experiences in the larger healthcare system as well. Future research should explore the dynamics of patient-provider interactions among PWID, including via cultural sensitivity training, alternatives to traditional healthcare structures, and focus on multi-level stigma interventions aimed at altering the attitudes, biases, and actions of individuals within the healthcare system as a whole.

## Data Availability

The data analyzed in the current study are not publicly available due to participant confidentiality but are available in de-identified forms from the corresponding author upon reasonable request.

## Abbreviations

ER: Emergency room
HCV: Hepatitis C virus
NYC: New York City
HIV: Human immunodeficiency virus
PrEP: Pre-exposure prophylaxis for HIV
PWID: People who inject drugs
PWUD: People who use drugs
SSP: Syringe service program
